# It's not easy being green: Comparing typical skin colouration among amphibians with colour abnormalities associated with chromatophore deficits

**DOI:** 10.1002/ece3.11438

**Published:** 2024-05-21

**Authors:** John Gould, Colin McHenry

**Affiliations:** ^1^ School of Environmental and Life Sciences University of Newcastle Callaghan New South Wales Australia

**Keywords:** amphibian, axanthism, colour mutation, hypomelanism, iridophore, melanophore, xanthophore

## Abstract

Amphibians can obtain their colour from a combination of several different pigment and light reflecting cell types called chromatophores, with defects in one or several of the cells leading to colour abnormalities. There is a need for better recording of colour abnormalities within wild amphibian populations, as this may provide baseline data that can be used to determine changes in environmental conditions and population dynamics, such as inbreeding. In this study, we provide records of several types of chromatophore deficiencies, including those involving iridophores, xanthophores and melanophores, among two Australian tree frog species; the green and golden bell frog, *Litoria aurea*, and the eastern dwarf tree frog, *L. fallax*. We explore these colour abnormalities in terms of the chromatophores that have likely been affected and associated with their expression, in combination with typical colour phenotypes, colour variations and colour changes for these species. We intend for our photographs to be used as a visual guide that addresses the need for more accessible information regarding the physical manifestation of different chromatophore defects among amphibians.

## Graphical abstract

Amphibians obtain their colour from several different pigment and light reflecting cells called chromatophores, with defects in one or several of the cells leading to colour abnormalities. We provide records of several types of chromatophore deficiencies, including those involving iridophores, xanthophores and melanophores, among two Australian tree frog species and compare these to typical phenotypes of these species and similar aberrations in other amphibians. We intend for our work to be used as a visual guide that addresses the need for more accessible information regarding the physical manifestation of different chromatophore defects among amphibians.

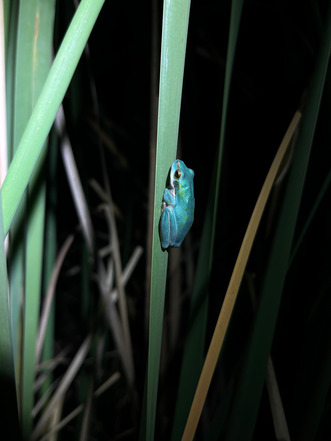

## 1 INTRODUCTION

Amphibians are one of the most colour diverse animal groups, with skin and eye colouration playing important roles in the life history of species and individual fitness (Glaw & Vences, 1997; Hoffman & Blouin, 2000). Species‐specific skin colour patterns are used in intraspecific communication, such as sex recognition (Sztatecsny et al., 2012), displays of dominance in male–male competition (Duellman & Trueb, 1994), and providing information regarding mate quality (Gomez et al., 2009), and for interspecific visual communication, such as providing warning signals to alert predators of potential toxicity (aposematic colouration; Noonan & Comeault, 2009; Poulton, 1890). Other roles of skin colouration include camouflage to hide from predators (Rojas, 2017), and for thermal regulation (Trullas et al., 2007). Similarly, eye colouration has several proposed adaptive functions, such as for aposematic mimicry or to blend into surrounding skin colour patterns (Glaw & Vences, 1997; Kuchta et al., 2008). The diversity of colour patterns found among amphibians is the direct result of different selecting pressures, leading to a range of typical phenotypes for each species, including colour variations within populations (colour polymorphism; Hoffman & Blouin, 2000) and between populations experiencing different environmental conditions (Wilkens, 1988).

Amphibians obtain their colour via light interacting with a layered arrangement of three main types of specialised cells called chromatophores that exist within the dermis skin layer; two of which contain pigments that absorb light and one that contains reflective platelets that reflect or scatter light (Bechtel, 1995; Duellman & Trueb, 1994). Each chromatophore unit consists of a xanthophore (containing yellow pigments that absorb short wavelengths such as blue‐violet), which lies above an iridophore (containing reflective platelets that reflect or scatter the remaining short wavelengths yellow‐green), and a melanophore (containing dark melanin pigment that absorbs long wavelengths such as red‐orange) below (Bagnara et al., 1968; Bagnara & Hadley, 1973); layering differences have been detected in some species (e.g., Kobelt & Linsenmair, 1986). A visual representation of this unit can be found in Vitt and Caldwell (2013). It is the combination of all three chromatophores that give many frogs their stereotypical green colouration, as intermediate wavelengths (green) pass back through the skin (Bagnara & Hadley, 1973; Taylor & Bagnara, 1972). Beyond the chromatophore unit, melanocytes may also be present more superficially within the epidermis (Smith‐Gill et al., 1972), and structural materials such as collagen within the skin may directly interact with light and contribute to skin colouration (Bagnara et al., 2007). In the absence of chromatophores, species with translucent skin may also attain colour from extracellular pigments (Franco‐Belussi et al., 2016; Taboada et al., 2020). The colouration of many amphibians is plastic, with stress, environmental background colour and temperature triggering pathways, notably hormonal, that allow individuals to adjust their phenotypic expression (Nilsson et al., 2013); an example is colour change that can occur rapidly via movement of pigments within chromatophore cells (Nielsen, 1978). The effects of chromatophore combinations and plasticity in their contribution to skin appearance highlight the complexity of colouration among amphibians.

Given its role in how individuals visually interact with their environment, colouration is under strong selecting forces that should lead to the rapid elimination overtime of deleterious colours or patterns (Andren & Nilson, 1981). Aberrant colourations are rare in natural populations (Henle & Dubois, 2017; Hoffman & Blouin, 2000), usually the result of genetic or environmental factors that affect the development, pigmentation, density and distribution of one or several of these cells (Duellman & Trueb, 1994). They may occur as part of the natural gene pool (Mitchell & Church, 2002), or they can be caused by environmental pollution (Henle & Dubois, 2017). Human disturbances such as habitat fragmentation can increase the visibility of rare alleles in affected populations via population isolation and inbreeding depression (Bensch et al., 2000; Vershinin, 2004). No matter what their cause, these colour variations may be harmful for individuals if it causes them to be more susceptible to visually oriented predators—affecting cryptic species if it increases their conspicuousness (Childs Jr, 1953), or aposematic species if it decreases the strength of warning signals; for amphibian species that are primarily nocturnal the impact of abnormal colouring may be lessened due to the poor colour vision of most predator animals at night (Sillman et al., 1997). Variation in colour may also reduce reproductive potential if it reduces an individual's detectability by or their visual appeal to the opposite sex, especially since many amphibians show excellent colour vision in both dark and light environments (Aho et al., 1993; Cummings et al., 2008). Yet, colour mutations can sometimes be advantageous for animals, such as by increasing the inconspicuousness of individuals within human‐altered environments (Askew et al., 1971), or by causing predators to be reluctant to attack visually novel prey (neophobia; Exnerová et al., 2006), or have neutral impacts on fitness, particularly beyond early life stages (Bensch et al., 2000). Further studies are warranted showing the occurrence rate and potential impacts of abnormal colouring among amphibians.

Understanding the ecological implications of colour mutations relies in part on measuring their occurrence in wild populations. Potentially, changes in the occurrence rates of such abnormalities can be used as a rapid, visual means of monitoring populations for threats, such as inbreeding depression or environmental pollution. Indeed, colour aberrations may be associated with other deleterious mutations that may not be so easily detected at a distance (Browder, 1972; Sanabria et al., 2010). Unusual colouring or colour changes may also be an indication of the condition of individuals, such as changes in skin iridescence related to hydration (Kobelt & Linsenmair, 1992). Colour variants can also reveal information about the distribution of chromatophores of target species, which may not be visually apparent in individuals with typical colouration (Turner, 2017). While notes regarding observations of abnormal colouration are widespread among amphibians, occurrence rates within populations are not always provided, preventing the detection of changes in their occurrence from base rates that may provide valuable insights into the functioning of amphibian populations.

In this study, we present several different types of abnormal colouration that are the result of chromatophore deficiencies, detected during extensive field surveys of sympatric populations of two Australian tree frog species. Our primary objectives were to provide occurrence rates of each colour aberration and to compare these aberrations with typical colour phenotypes, adjustments and variability found within these species. We have inferred the chromatophore cell types that are likely affected for each aberration detected based on skin colouration and compared them with similar aberrations in other amphibians as part of a mini review. We have provided high quality photographs of wild individuals with both typical and abnormal colourations to be used as visual guides for understanding the physical manifestation of different chromophore defects among amphibians.

## 2 FIELD METHODS

All case studies used in this study are derived from observations made on Kooragang Island, NSW, Australia (32.85837°S and 151.72480°E). This island is situated at the mouth of the Hunter River and has undergone widespread modification from agricultural and industrial activities, as well as recent remediation involving pond construction, resulting in the presence of semi‐natural and artificial freshwater ponds interspersed by mangrove creeks and saltmarsh (Beranek et al., 2020; Gould et al., 2024).

Across four consecutive years from 2020 to 2024, we surveyed 80–90 freshwater ponds on the island between September and April to monitor one of the largest extant populations of the threatened green and golden bell frog (*Litoria aurea*). Each pond was surveyed at night by two or more researchers with headtorches for a maximum of 30 min by wading around the waterline and throughout emergent aquatic vegetation. During these surveys, we also recorded the presence of a sympatric species, the eastern dwarf tree frog (*L. fallax*) and any instances of colour abnormalities in both species.

## 3 BLUE SKIN AND XANTHOPHORE DEFICIENCY

### 3.1 Amphibians with naturally blue skin

Few amphibians have blue skin colours or patterns that are considered typical. Exceptions include some morphs of the poison dart frog (*Dendrobates tinctorius*), where both sexes are almost entirely blue (d'Orgeix et al., 2019), the moor frog (*Rana arvalis*), which shows dynamic sexual dichromatism as males turn blue during breeding periods (Rojas, 2017), and the Indian bullfrog (*Hoplobatrachus tigerinus*) in which breeding males exhibit blue vocal sacs (AmphibiaWeb, 2010; Thongproh et al., 2022). For these species, the blue colouring may be used as a visual cue for sex recognition (Sztatecsny et al., 2012), or as a warning sign (aposematism) to prevent predation (Caro & Ruxton, 2019; Rojas & Endler, 2013). Blue skin colours are caused by the reflectance of light from iridophores in the presence of melanophores below, but in the absence of overlying xanthophores (Bagnara et al., 2007; Rodríguez et al., 2020).

#### 3.1.1 Case study—*Litoria aurea*

**FIGURE 1:**
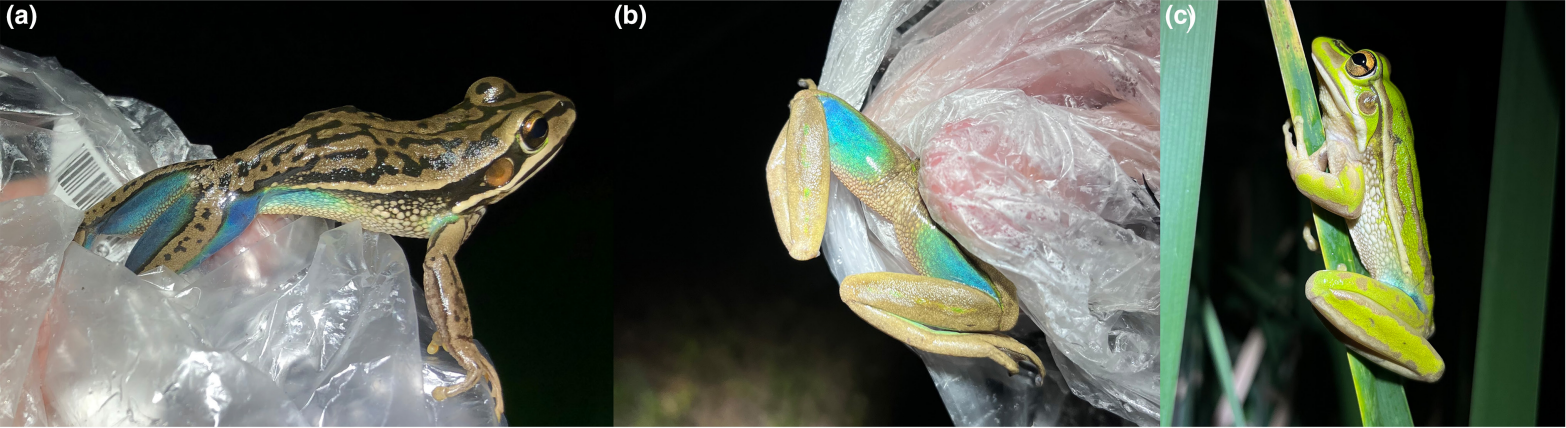
Blue flash colouration exhibited by adult male and female green and golden bell frogs, *Litoria aurea*, from Kooragang Island, NSW, Australia. The blue skin is (a) and (b) expressed along the inner thighs and armpits and (c) hidden when individuals are in a resting position.

Among Australian amphibians, there are no species that are naturally blue across a majority of their body surfaces (Anstis, 2007), yet some do show blue markings in select body regions. The most notable include those within the bell frog species group, such as *L. aurea*. Both sexes of this species have vivid turquoise blue along the thighs, running into the lower sides, as well as the armpits (Figure 1). The adaptive purpose of this blue marking, if any, remains undetermined. Its presence in both sexes suggests that its occurrence is unlikely to be for intraspecific communication but it may be form of hidden aposematism (flash colouration) exhibited against visual orientated predators during escape or posturing (Ferreira et al., 2019). Consistent with that hypothesis, the blue coloured skin is almost entirely hidden when these frogs are resting in a neutral position (Figure 1).

### 3.2 Axanthism among amphibians

Blue skin colour aberrations have been recorded globally among amphibians (Henle & Dubois, 2017), particularly species in the family Randiae and more often in frogs from North America (Berns & Uhler, 1966; Jablonski et al., 2014). Yet, it has been recorded in only a few Australian species and rarely photographed. Exceptions include a completely blue green tree frog (*L. caerulea*) and a partially blue Barrington Tops tree frog (*L. barringtonensis*; Robinson, 2022). These aberrations are most likely the result of mutations causing a lack of the carotenoid‐bearing xanthophores or their pigment and leading to axanthism (Bagnara et al., 1978; Berns & Narayan, 1970; Browder, 1968). This prevents the filtering of short wavelengths of light that are scattered from the iridophore cells within the chromatophore unit, which results in the expression of blue skin across affected surfaces where green skin would have been expressed, typically the dorsum (Berns & Narayan, 1970; Miller et al., 2018). Another potential cause of blue skin aberrations is the absence of both xanthophores and iridophores within the skin, with the presence of collagen leading to the scattering of blue light (Bagnara et al., 2007). However, we are not aware of visual comparisons of blue colouration caused by these two different processes.

Axanthism is a rare aberration, particularly among adults, which may be related to the reduced fitness of individuals if the abnormal colouring results in increased predation or is associated with other harmful mutations (Dubois, 1979; Jablonski et al., 2014). For example, the rate of axanthism in the green frog (*Lithobates clamitans*), has been recorded at 0.2–0.3% (Berns & Uhler, 1966). Disparities in the frequency of its occurrence between species and regions may be an artefact of research intensity (Jablonski et al., 2014), yet there may be a higher predisposition for its occurrence which is species specific.

#### 3.2.1 Case study—*Litoria aurea*

**FIGURE 2:**
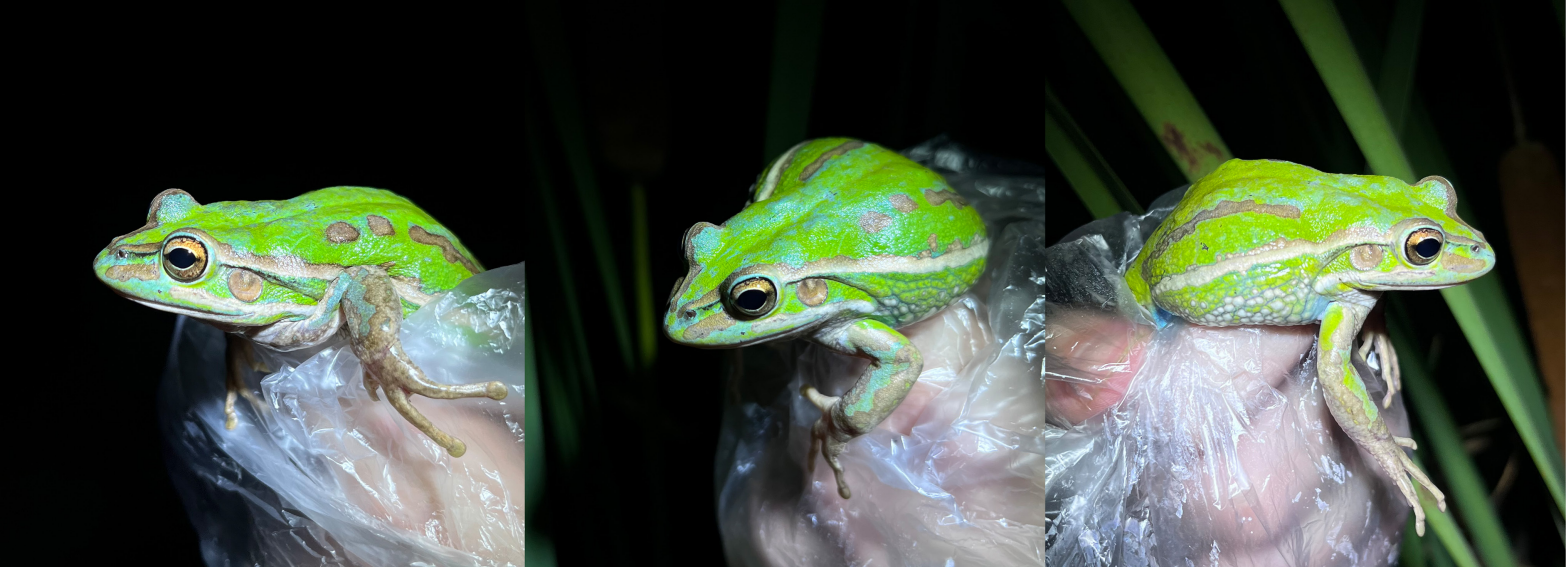
Partial axanthism in an adult female green and golden bell frog, *Litoria aurea*, captured on Kooragang Island, NSW, Australia. The individual shows the typical green dorsal colouration with patches of light blue across the face, back and front limbs.

We recently made the first observation of partial axanthism in *L. aurea*. In December 2023, an adult *L. aurea* female showing blue skin patches was captured in a constructed wetland pond within a *Typha* stand on Kooragang Island (32.86976° S, 151.73119° E). An opaque, aquamarine colouration was confined to small patches on the face, including the nose, upper eyelids and upper lip, as well as the dorsum and forelimbs (Figure 2). Normal colouration was found across remaining areas of dorsum (light green), as well as the vent (opaque white), irises (metallic gold with black venation) and thighs (opaque turquoise). The individual was partially gravid and showed good condition (as noted by the large size of the body relative to the head). This axanthism differs from the typical blue flash thigh colouration that also occurs in this species, with the relatively greenish hue of the abnormal blue skin suggesting that there was only a deficit in xanthophores or their pigment as opposed to a complete absence. Across four consecutive years (2020–2023), we detected only one partially axanthic blue *L. aurea* out of 7193 observations (0.01%).

#### 3.2.2 Case study—*Litoria fallax*

**FIGURE 3:**
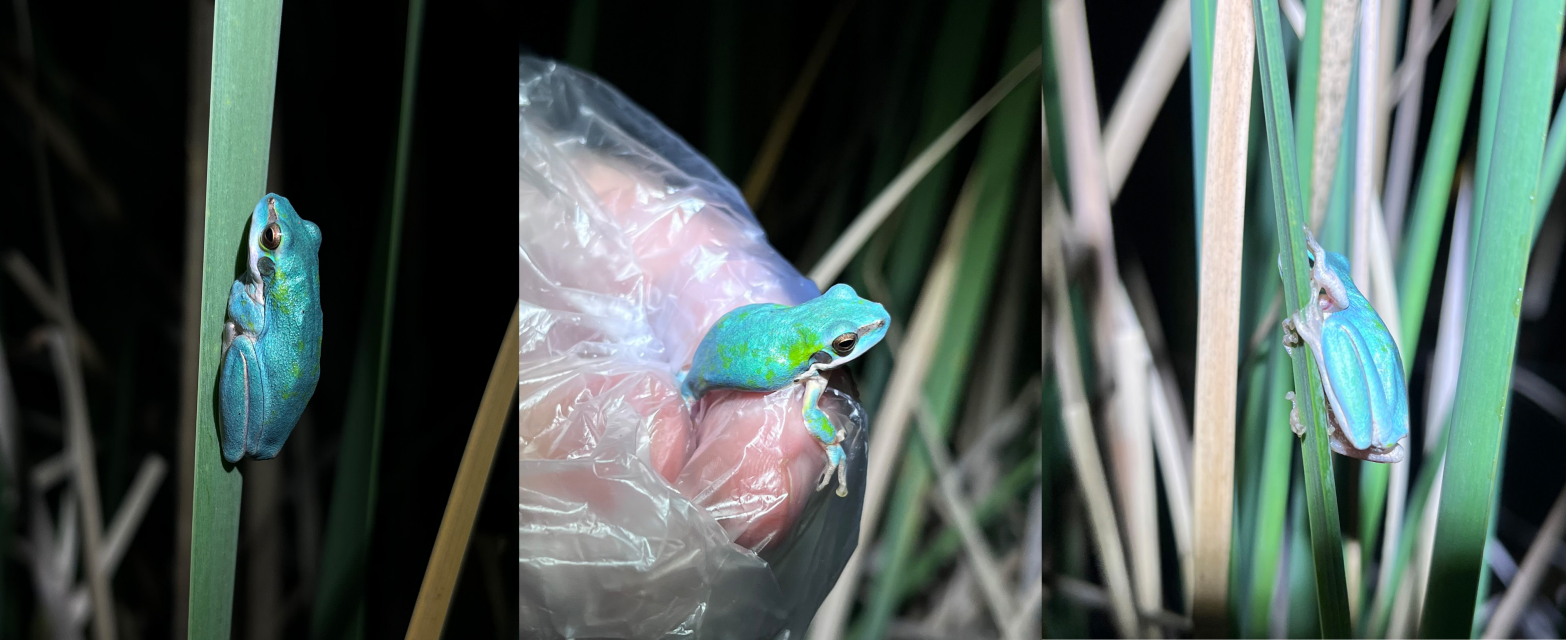
Near complete axanthism in an adult eastern dwarf tree frog, *Litoria fallax*, captured on Kooragang Island, NSW, Australia. The individual shows only a few patches of typical green dorsal colouration.

We recently made the first observation of near complete axanthism in *L. fallax*. In December 2023, we observed a blue adult of unknown sex within a constructed pond on Kooragang Island (32.86481° S, 151.73507° E), resting on a *Baumea* plant 60 cm above water. The frog was almost entirely opaque, cerulean blue, including the face, dorsum, flanks and limbs (Figure 3), suggesting a deficit in xanthophores. Freckling of green skin colouration typical for this species was apparent on the dorsum, indicating normal functioning of all chromatophores in these specific areas, including all three chromatophore cell types. The ventral surface showed normal colouration (opaque white), as well as the irises (melic gold with black venation), indicating that all other chromatophores besides xanthophores were unaffected. Across four consecutive years (2021–2023), we detected only one axanthic blue *L. fallax* out of 12,350 observations (0.008%).

**FIGURE 4:**
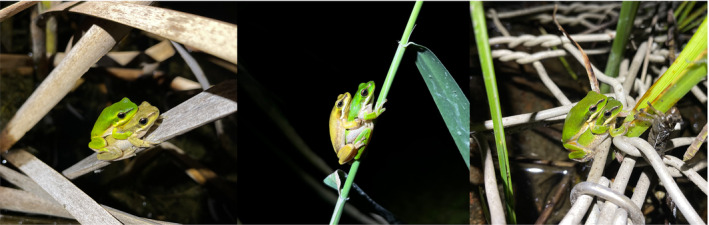
Variations in dorsal skin colouration in the eastern dwarf tree frog, *Litoria fallax*, that is considered part of its normal phenotype. Both adult males and females photographed in amplexus range from dark green to light brown.

The total blue colouration of all dorsal surfaces in this *L. fallax* individual differed from the typical colouration of this species. In particular, *L. fallax* typically show dorsal skin that ranges from bronze to dark green in both sexes (Figure 4), with no expression of blue colouration in any skin region.

## 4 YELLOW SKIN AND MELANOPHORE DEFICIENCY

### 4.1 Amphibians with typical yellow colouration

Yellow colouration is part of the typical phenotype of some amphibians and forms via the presence of xanthophores when melanophores are either absent or if the melanosome organelles storing the melanin pigment within melanophores are aggregated to prevent xanthophore concealment (Kindermann & Hero, 2016a). The most noticeable example of the latter occurs in male eastern stony creek frogs (*Litoria wilcoxii*), which undergo rapid physiological colour change from brown to opaque yellow that occurs after mate selection and amplexus has been initiated (Kindermann & Hero, 2016b); a similarly rapid colour change has been exhibited in adult male *Bufo luetkenii* (Doucet & Mennill, 2010). This rapid dichromatism in *L. wilcoxii* may be used as a visual cue to deter conspecific males and is under neuro‐hormonal control. Epinephrine induced pathways cause melanosomes within melanophores to become aggregated within the centre of these cells, thus allowing for the exposure of the yellow xanthophores on top that are usually covered by the melanophore pigment when it is dispersed (Kindermann et al. 2014; Kindermann & Hero, 2016a). In this species, iridophores are also absent from the yellow skin (Kindermann & Hero, 2016a) but in other species yellow skin contain both xanthophores and iridophores (e.g., *Ambystoma maculatum*; Taylor & Bagnara, 1972). We are unsure how the presence of iridophores influences the expression of yellow skin colouration except to brighten the xanthophore pigment and/or increase the opaqueness of the skin (Bagnara, 1966).

#### 4.1.1 Case study—*Litoria aurea*

**FIGURE 5:**
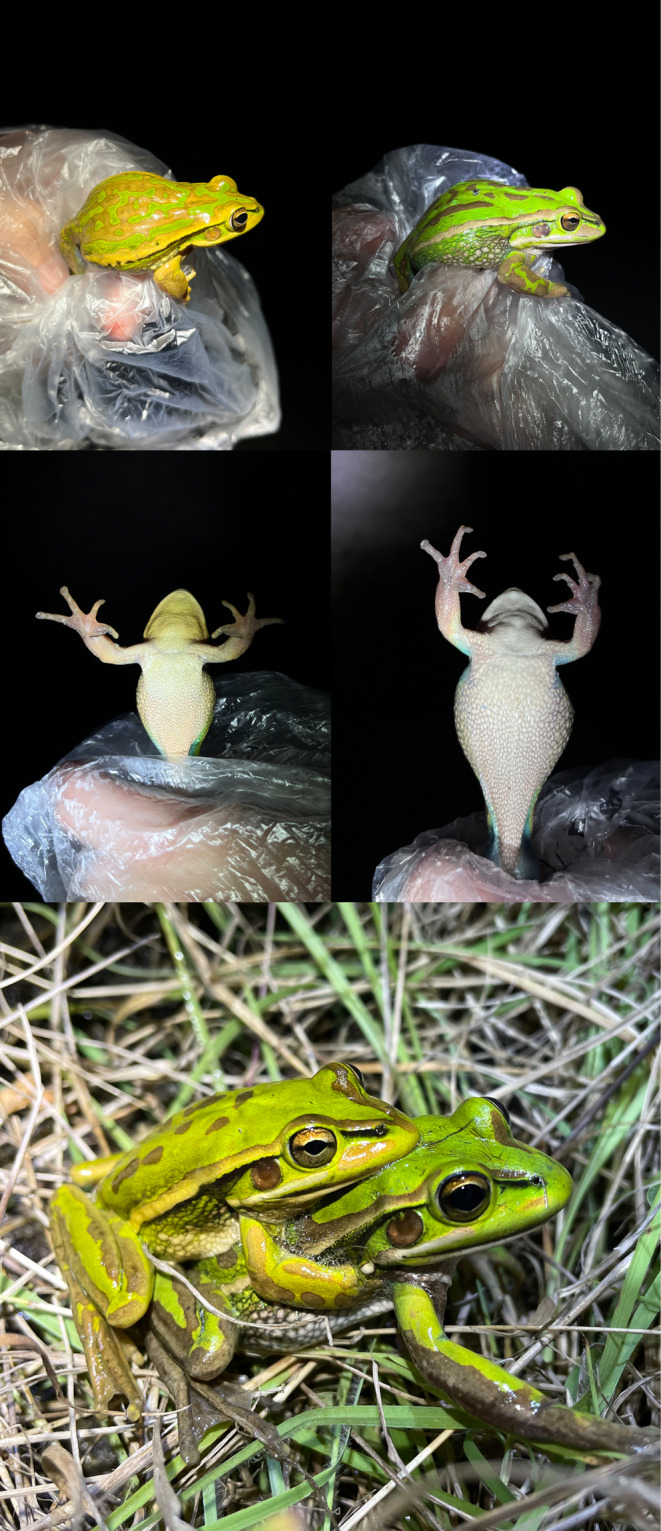
Dichromatism in adult green and golden bell frogs, *Litoria aurea*, during the breeding season. Male (left) shows yellowing of dorsal and throat surfaces while female (right) has retained green dorsal surfaces and white throat. The yellowing of the male's skin compared with that of the female can be seen in the mating pair in amplexus.

We have observed dynamic sexual dichromatism in *L. aurea* during the breeding season, with male individuals exhibiting relatively more yellowing of green dorsal skin and white banding, as well as discoloured yellowish throats when compared to females that possess the typical green dorsal skin colouration with white banding and white throats (Figure 5). This yellowing in *L. aurea* appears to be extended for the duration of the breeding season (several weeks or more) and may be under steroid‐hormonal control (e.g., Richards, 1982), particularly elevated testosterone levels that causes either a (i) slow, temporary morphological colour change via the increase in xanthophore pigment synthesis (Kindermann et al. 2014), or (ii) a slow, temporary physiological colour change via the dispersion of xanthophore pigment organelles simultaneous to the aggregation of melanosomes within the melanophores (Tang et al., 2014). As yellowing in males occurs over areas of white skin for extended periods with relatively little difference in brown dorsal patterning between the sexes, we hypothesise that increased testosterone leading to xanthophore pigment synthesis is the primary cause for dorsal yellowing in *L. aurea* as opposed to a rapid change in the aggregation of melanosomes within melanophores as seen in *L. wilcoxii*, Seasonal changes in testicular structure and consequently hormones have also been associated with the cyclic hypertrophy/darkening and regression/lightening of male nuptial pads (Lofts, 1964; Lynch & Blackburn, 1995), a secondary sex characteristic used to assist with grasping females during amplexus. It remains to be determined whether this extended sexual dichromatism seen in *L. aurea* has an adaptive function in terms of intraspecific visual communication (mate choice or male–male competition) or is simply an artefact of hormonal changes that occur with reproductive cycles.

### 4.2 Hypomelanism among amphibians

Conditions that lead to deficiencies or the absence in melanin pigment are referred to as hypomelanism. This includes albinism (total amelanism), which is an autosomal recessive gene inheritance that results in the complete or near complete absence in biosynthesis of melanin pigment across all pigmented tissues including the eyes (both the iris and retina) (Lunghi et al., 2017; Sazima, 1974), and leucism which affects the skin and irises but not the retinas. In the absence of melanin (hypomelanism), amphibians may still express colouring due to the presence of non‐melanin pigment cell types in the skin that are not affected (Smith‐Gill et al., 1972). For example, fire salamanders (*Salamandra salamandra*) have black skin that is melanin rich with likely few other chromatophore cell types present, with yellow patches that are low in melanin but contain high abundances of xanthophores (and possibly iridophores). Albino individuals of this species express flesh/pink skin and eyes due to the absence of melanin in areas that are typically black but continue to express yellow skin patches that are the result of the unaffected presence of xanthophores (Lunghi et al., 2017).

#### 4.2.1 Case study—*Litoria aurea*

**FIGURE 6:**
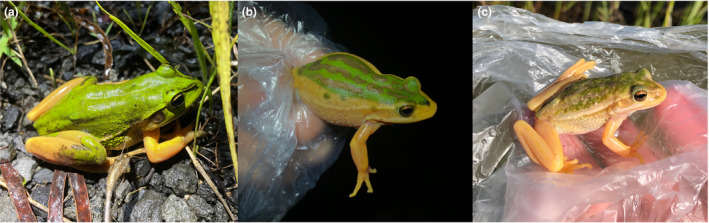
Partial hypomelanism of dorsal skin surfaces in adult green and golden bell frogs, *Litoria aurea*, from a wild population on Kooragang Island, NSW, Australia. Individuals show melanin deficiency across (a) the limbs, (b) the limbs and flanks and (c) all dorsal surfaces.

Across four consecutive breeding seasons, we detected three adult *L. aurea* individuals out of 7193 observations (0.04%) exhibiting partial hypomelanism to varying extents across Kooragang Island (Figure 6). These individuals exhibited abnormal, opaque yellowing of dorsal skin that is typically opaque green, suggesting the loss of melanin but the maintenance of other chromatophores, namely iridophores and xanthophores. Depigmentation occurred (i) only on the extremities, including completely on the forelimbs and partially on the hindlimbs, with no other skin sections affected, (ii) the front of the face, flanks and extremities, with the central dorsum of the back and head retaining normal skin colouration and (iii) almost all skin surfaces except for partial, mottled green colouration of the dorsum (Figure 6). Individuals with their head dorsum affected showed a noticeable lack of black banding running between the nose and eyes, further evidence of melanin deficiency. The affected skin was also opaque yellow in appearance when compared to ventral skin that remained opaque white, the latter of which is typical for this species. All individuals possessed normal pigmentation of the iris, which was metallic gold with black venation and surrounded by a rim of black, and black retinas, indicating no other chromatophores besides melanin pigment cells in the skin were affected. The yellow colouration caused by hypomelanism differs from the typical yellowing that is also present among adult males of this species during the breeding season. The rate of hypomelanism we detected in *L. aurea* is much lower than for other species such as Zeus' robber frog (*Eleutherodactylus (Syrrophus) zeus*) where 26% of detected individuals showed abnormal white patches suggestive of partial leucism (García‐Padrón & Bosch, 2019).

## 5 PALE SKIN AND IRIDOPHORE DEFICIENCY

### 5.1 Amphibians with naturally white or translucent skin

Skin transparency, where light can easily pass through, or translucency, where light can partially pass through, are known to be the natural condition of several amphibians. This includes glass frogs, which possess almost totally transparent ventral surfaces that may be used for camouflage (Barnett et al., 2020; Rudh & Qvarnström, 2013). ‘See‐through’ skin results from the low concentration or lack of all chromatophores types within the skin. This may lead to the expression of skin translucency to varying extents, or true transparency where internal blood vessels, bones and organs are visible (Bruni et al., 2020; Sumida et al., 2016). Increased skin transparency can also occur when melanosomes are aggregated within melanophores in sections of skin where it is the primary chromatophore present (Nilsson et al., 2013).

In contrast, opaque white or silver skin among amphibians occurs when iridophores are the only chromatophore type present (Bagnara et al., 2007; Fernandez & Bagnara, 1993; Rudh & Qvarnström, 2013), or when melanophores are also present but their pigment is aggregated to prevent the concealment of the iridophores (Fernandez & Bagnara, 1993), or when there is an increase in the abundance of iridophores that creates a highly reflective layer above other chromatophores also present (Kobelt & Linsenmair, 1986). High skin reflectance leading to white skin colouration is dictated by the abundance of iridophores in the skin, their thickness and the arrangement of light reflecting platelets within these cells (Kobelt & Linsenmair, 1992). For many amphibians, lighter skin colours, including opaque white, are commonly expressed over ventral surfaces while darker and colourful skin is expressed across the dorsum; possibly as a form of crypsis in land animals (Rowland et al., 2008). This spatial separation of skin colouration in amphibians is at least partly caused by the presence of a melanisation inhibiting factor (MIF) specifically in ventral skin that leads to a lack of melanophore presence and promotes iridophore localisation (Bagnara & Fukuzawa, 1990; Fukuzawa et al., 1995).

In addition to producing opaque white skin, iridophores can also result in iridescent colours, producing ‘glittery’ or metallic silver and copper/gold to green (Kobelt & Linsenmair, 1992); these are commonly expressed in the iris of many amphibians above a background of melanophores (Duellman & Trueb, 1994; Glaw & Vences, 1997). In the skin, the presence of iridophores has also been suggested to brighten the xanthophore pigment in the absence of melanophores (Bagnara, 1966).

### 5.2 Lack of iridophores

Skin transparency or translucency can be a chromatic aberration, resulting from genetic mutations that cause a deficiency in all skin chromatophores within affected areas (Bruni et al., 2020; Sumida et al., 2016). If multiple chromophores are present in the skin, then transparency or translucency can only occur if all chromatophore types are deficient. Skin transparency aberrations have been observed in several frogs. For example, total deficiency in iridophores across typically opaque white ventral surfaces has been recorded in an Australian tree frog, *L. rothii* (Turner, 2017), leading to transparent skin, while partial deficiency in chromatophores leading to transparent skin patches has been detected in Po's tree frog (*Hyla perrini*; Bruni et al., 2020).

#### 5.2.1 Case study—*Litoria aurea*

**FIGURE 7:**
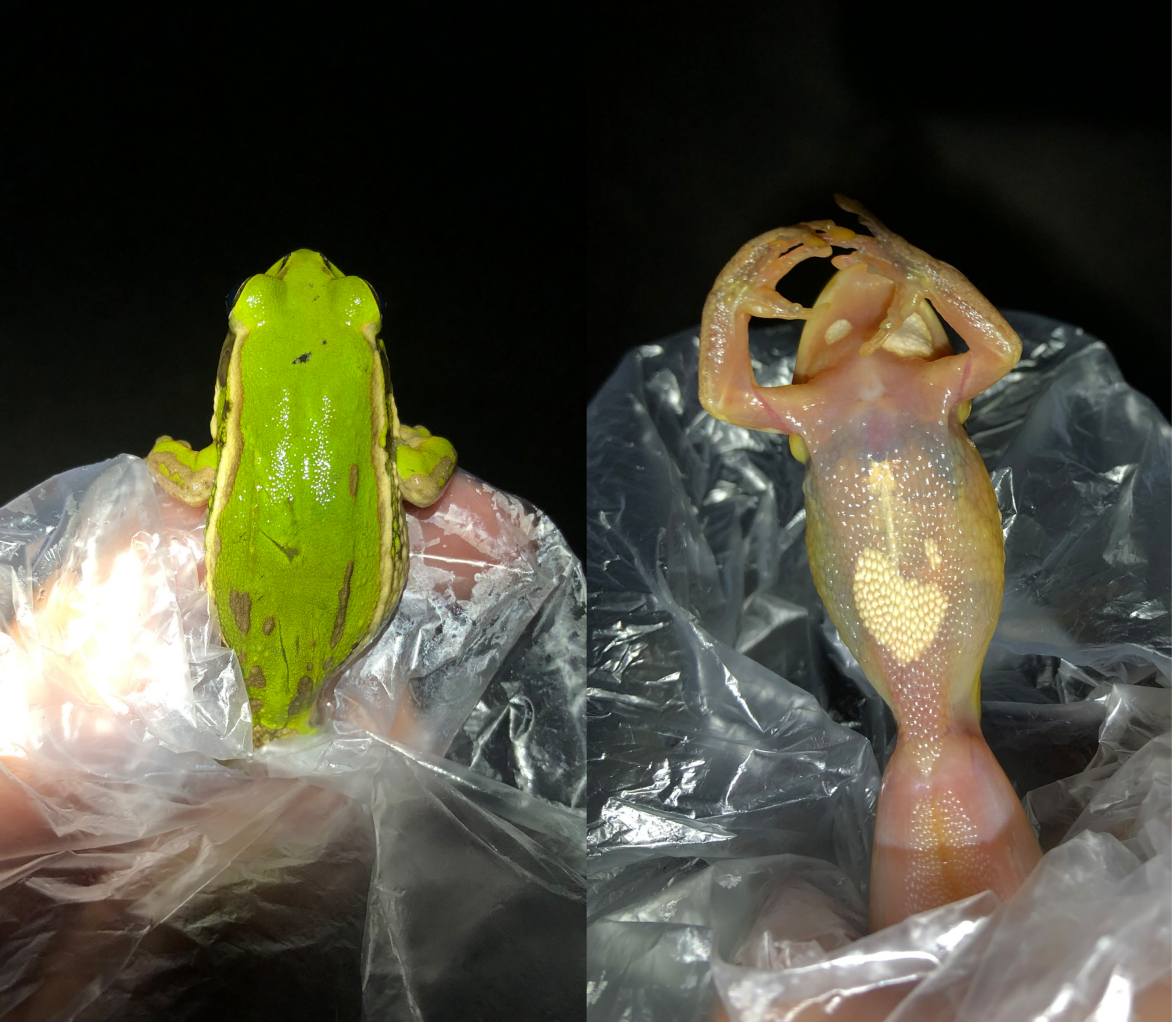
Ventral skin translucency detected in an adult green and golden bell frog, *Litoria aurea*, from Kooragang Island, NSW. Australia. The typical ventral skin colour for this species is opaque white caused by light scattering from a layer of iridophores, with skin translucency indicating a deficiency in this chromatophore. The individual displays typical green dorsal colouration with brown patterning and white banding.

In February 2021, we observed an adult male *L. aurea* with relatively translucent skin across most of its ventral surface in a constructed pond of Kooragang Island (32.87179° S, 151.74016° E, Figure 7). This suggests a near total lack of chromatophores; the vent of this species is typically opaque white, indicating that iridophores should typically be present and xanthophores and melanophores typically absent. Internal tissues and blood vessels were apparent but not well defined through this skin, potentially due to other structures within the skin leading to some light scattering or the presence of a small number of iridophores. Three opaque, white patches of skin consistent with typical ventral colouration caused by the presence of iridophores were located in the middle of its vent and over smaller sections of its throat, indicating piebald‐like deficiency in chromatophores. The *L. aurea* dorsum showed normal skin colouration, consisting of a background of green skin with gold markings, indicating the presence of all chromophores at typical levels in this region (Figure 7). We have only detected translucency in one out of 7193 observations (0.01%) across four consecutive breeding seasons.

## 6 CONCLUSION

The occurrence of colour anomalies within wild populations are rare and while they can occur naturally by chance, they may also be an indication of inbreeding processes, exposure to pollution or the effects of disease. Long term population studies should thus record changes in the frequency of each type of colour anomaly, as an effective visual means of monitoring populations and detecting the consequence of changes that may not be immediately apparent using typical monitoring techniques. We hope that our records of colour abnormalities in wild amphibian populations when compared to normal colour phenotypes can be used as a guide to assist other projects in explaining similar colour mutations.

## AUTHOR CONTRIBUTIONS

**John Gould:** Conceptualization (lead); investigation (equal); methodology (lead); visualization (lead); writing – original draft (lead); writing – review and editing (equal). **Colin McHenry:** Investigation (equal); supervision (lead); writing – original draft (supporting); writing – review and editing (equal).

## CONFLICT OF INTEREST STATEMENT

The authors declare no conflicts of interest.

## Data Availability

There is no data associated with this paper.

## References

[ece311438-bib-0001] Aho, A.‐C. , Donner, K. , Helenius, S. , Larsen, L. O. , & Reuter, T. (1993). Visual performance of the toad (*Bufo bufo*) at low light levels: Retinal ganglion cell responses and prey‐catching accuracy. Journal of Comparative Physiology A, 172, 671–682. 10.1007/BF00195393 8350284

[ece311438-bib-0002] AmphibiaWeb . (2010). Hoplobatrachus tigerinus: Indus Valley bullfrog. University of California.

[ece311438-bib-0003] Andren, C. , & Nilson, G. (1981). Reproductive success and risk of predation in normal and melanistic colour morphs of the adder, *Vipera berus* . Biological Journal of the Linnean Society, 15, 235–246.

[ece311438-bib-0004] Anstis, M. (2007). Frogs and tadpoles of Australia. New Holland Publishers.

[ece311438-bib-0005] Askew, R. , Cook, L. , & Bishop, J. (1971). Atmospheric pollution and melanic moths in Manchester and its environs. Journal of Applied Ecology, 8, 247–256.

[ece311438-bib-0006] Bagnara, J. (1966). Cytology and cytophysiology of non‐melanophore pigment cells. In G. Bourne & J. Danielli (Eds.), International review of cytology (pp. 173–205). Academic Press. 10.1016/s0074-7696(08)60801-3 5337298

[ece311438-bib-0007] Bagnara, J. T. , Fernandez, P. J. , & Fujii, R. (2007). On the blue coloration of vertebrates. Pigment Cell Research, 20, 14–26. 17250544 10.1111/j.1600-0749.2006.00360.x

[ece311438-bib-0008] Bagnara, J. T. , Frost, S. K. , & Matsumoto, J. (1978). On the development of pigment patterns in amphibians. American Zoologist, 18, 301–312.

[ece311438-bib-0009] Bagnara, J. T. , & Fukuzawa, T. (1990). Stimulation of cultured iridophores by amphibian ventral conditioned medium. Pigment Cell Research, 3, 243–250. 2095576 10.1111/j.1600-0749.1990.tb00296.x

[ece311438-bib-0010] Bagnara, J. T. , & Hadley, M. E. (1973). Chromatophores and color change‐ The comparative physiology of animal pigmentation, chromatophores and colour change. Prentice‐Hall.

[ece311438-bib-0011] Bagnara, J. T. , Taylor, J. D. , & Hadley, M. E. (1968). The dermal chromatophore unit. The Journal of Cell Biology, 38, 67–79. 5691979 10.1083/jcb.38.1.67 PMC2107474

[ece311438-bib-0012] Barnett, J. B. , Michalis, C. , Anderson, H. M. , McEwen, B. L. , Yeager, J. , Pruitt, J. N. , Scott‐Samuel, N. E. , & Cuthill, I. C. (2020). Imperfect transparency and camouflage in glass frogs. Proceedings of the National Academy of Sciences, 117(12), 885–890. 10.1073/pnas.1919417117 PMC7293656 32457164

[ece311438-bib-0013] Bechtel, H. B. (1995). Reptile and amphibian variants: Colour, patterns and scales. Krieger Publishing Company.

[ece311438-bib-0014] Bensch, S. , Hansson, B. , Hasselquist, D. , & Nielsen, B. (2000). Partial albinism in a semi‐isolated population of great reed warblers. Hereditas, 133, 167–170. 11338429 10.1111/j.1601-5223.2000.t01-1-00167.x

[ece311438-bib-0015] Beranek, C. T. , Clulow, J. , & Mahony, M. (2020). Wetland restoration for the threatened green and golden bell frog (*Litoria aurea*): Development of a breeding habitat designed to passively manage chytrid‐induced amphibian disease and exotic fish. Natural Areas Journal, 40, 362–374.

[ece311438-bib-0016] Berns, M. W. , & Narayan, K. S. (1970). An histochemical and ultrastructural analysis of the dermal chromatophores of the variant ranid blue frog. Journal of Morphology, 132, 169–179.

[ece311438-bib-0017] Berns, M. W. , & Uhler, L. D. (1966). Blue frogs of the genus *Rana* . Herpetologica, 22, 181–183.

[ece311438-bib-0018] Browder, L. W. (1968). Pigmentation in *Rana pipens*: I. Inheritance of the speckle mutation. Journal of Heredity, 59, 163–167. 5703372 10.1093/oxfordjournals.jhered.a107674

[ece311438-bib-0019] Browder, L. W. (1972). Genetic and embryological studies of albinism in *Rana pipiens* . Journal of Experimental Zoology, 180, 149–155. 4537230 10.1002/jez.1401800202

[ece311438-bib-0020] Bruni, G. , Di Mitri, A. , Grecchi, L. , & Di Nicola, M. R. (2020). “Translucent” colour aberrations in *Bufotes balearicus* (Anura: Bufonidae) and *Hyla perrini* (Anura: Hylidae) from Italy. Herpetology Notes, 13, 57–60.

[ece311438-bib-0021] Caro, T. , & Ruxton, G. (2019). Aposematism: Unpacking the defences. Trends in Ecology & Evolution, 34, 595–604. 31027839 10.1016/j.tree.2019.02.015

[ece311438-bib-0022] Childs, H. E., Jr. (1953). Selection by predation on albino and normal spadefoot toads. Evolution, 7, 228–233.

[ece311438-bib-0023] Cummings, M. E. , Bernal, X. E. , Reynaga, R. , Rand, A. S. , & Ryan, M. J. (2008). Visual sensitivity to a conspicuous male cue varies by reproductive state in *Physalaemus pustulosus* females. Journal of Experimental Biology, 211, 1203–1210. 18375844 10.1242/jeb.012963

[ece311438-bib-0024] d'Orgeix, C. A. , Hardy, D. , Witiak, S. M. , Robinson, L. R. , & Jairam, R. (2019). The blue dyeing poison‐dart frog, *Dendrobates tinctorius* (*Dendrobates azureus*, Hoogmoed 1969): Extant in Suriname based on a rapid survey. Amphibian and Reptile Conservation, 13, 259–264.

[ece311438-bib-0025] Doucet, S. M. , & Mennill, D. J. (2010). Dynamic sexual dichromatism in an explosively breeding Neotropical toad. Biology Letters, 6, 63–66. 19793736 10.1098/rsbl.2009.0604 PMC2817257

[ece311438-bib-0026] Dubois, A. (1979). Anomalies and mutations in natural populations of the *Rana* “esculenta” complex (Amphibia, Anura). Mitteilungen aus dem Zoologischen Museum in Berlin, 55, 59–87.

[ece311438-bib-0027] Duellman, W. , & Trueb, L. (1994). Biology of amphibians. John Hopkins University Press.

[ece311438-bib-0028] Exnerová, A. , Svádová, K. , Štys, P. , Barcalová, S. , Landová, E. , Prokopova, M. , Fuchs, R. , & Socha, R. (2006). Importance of colour in the reaction of passerine predators to aposematic prey: Experiments with mutants of *Pyrrhocoris apterus* (Heteroptera). Biological Journal of the Linnean Society, 88, 143–153.

[ece311438-bib-0029] Fernandez, P. J. , & Bagnara, J. T. (1993). Observations on the development of unusual melanization of leopard frog ventral skin. Journal of Morphology, 216, 9–15. 8496971 10.1002/jmor.1052160103

[ece311438-bib-0030] Ferreira, R. B. , Lourenço‐de‐Moraes, R. , Zocca, C. , Duca, C. , Beard, K. H. , & Brodie, E. D. (2019). Antipredator mechanisms of post‐metamorphic anurans: A global database and classification system. Behavioral Ecology and Sociobiology, 73, 69.

[ece311438-bib-0031] Franco‐Belussi, L. , Nilsson, S. H. , & De Oliveira, C. (2016). Internal pigment cells respond to external UV radiation in frogs. Journal of Experimental Biology, 219, 1378–1383. 26944494 10.1242/jeb.134973

[ece311438-bib-0032] Fukuzawa, T. , Samaraweera, P. , Mangano, F. T. , Law, J. H. , & Bagnara, J. T. (1995). Evidence that MIF plays a role in the development of pigmentation patterns in the frog. Developmental Biology, 167, 148–158. 7851638 10.1006/dbio.1995.1013

[ece311438-bib-0033] García‐Padrón, L. Y. , & Bosch, R. A. (2019). Anomalous colour in a Cuban cave‐dwelling frog: First record of piebaldism in *Eleutherodactylus zeus* (Anura: Eleutherodactylidae). Herpetological Bulletin, 157, 1–3.

[ece311438-bib-0034] Glaw, F. , & Vences, M. (1997). Anuran eye colouration: Definitions, variation, taxonomic implications and possible functions. Herpetologia Bonnensis, 1997, 125–138.

[ece311438-bib-0035] Gomez, D. , Richardson, C. , Lengagne, T. , Plenet, S. , Joly, P. , Léna, J.‐P. , & Théry, M. (2009). The role of nocturnal vision in mate choice: Females prefer conspicuous males in the European tree frog (*Hyla arborea*). Proceedings of the Royal Society B: Biological Sciences, 276, 2351–2358. 10.1098/rspb.2009.0168 PMC2690462 19324736

[ece311438-bib-0036] Gould, J. , Callen, A. , Beranek, C. , & McHenry, C. (2024). The only way is down: Placing amphibian ponds on plateaux protects against *Gambusia* colonization. Restoration Ecology. e14159.

[ece311438-bib-0037] Henle, K. , & Dubois, A. (2017). Studies on anomalies in natural populations of amphibians. Mertensiella, 25, 185–242.

[ece311438-bib-0038] Hoffman, E. A. , & Blouin, M. S. (2000). A review of colour and pattern polymorphisms in anurans. Biological Journal of the Linnean Society, 70, 633–665.

[ece311438-bib-0039] Jablonski, D. , Alena, A. , Vlček, P. , & Jandzik, D. (2014). Axanthism in amphibians: A review and the first record in the widespread toad of the *Bufotes viridis* complex (Anura: Bufonidae). Belgian Journal of Zoology, 144, 93–101.

[ece311438-bib-0040] Kindermann, C. , & Hero, J.‐M. (2016a). Pigment cell distribution in a rapid colour changing amphibian (*Litoria wilcoxii*). Zoomorphology, 135, 197–203.

[ece311438-bib-0041] Kindermann, C. , & Hero, J.‐M. (2016b). Rapid dynamic colour change is an intrasexual signal in a lek breeding frog (*Litoria wilcoxii*). Behavioral Ecology and Sociobiology, 70, 1995–2003.

[ece311438-bib-0042] Kindermann, C. , Narayan, E. J. , & Hero, J. M. (2014). The neuro‐hormonal control of rapid dynamic skin colour change in an amphibian during amplexus. PLoS One, 9, e114120. 25470775 10.1371/journal.pone.0114120 PMC4254939

[ece311438-bib-0043] Kobelt, F. , & Linsenmair, K. E. (1986). Adaptations of the reed frog *Hyperolius viridiflavus* (Amphibia, Anura, Hyperoliidae) to its arid environment. I. The skin of *Hyperolius viridiflavus nitidulus* in wet and dry season conditions. Oecologia, 68, 533–541. 28311709 10.1007/BF00378768

[ece311438-bib-0044] Kobelt, F. , & Linsenmair, K. E. (1992). Adaptations of the reed frog *Hyperolius viridiflavus* (Hyperoliidae) to its arid environment. Journal of Comparative Physiology B, 162, 314–326. 10.1007/BF00260758 1506488

[ece311438-bib-0045] Kuchta, S. R. , Krakauer, A. H. , & Sinervo, B. (2008). Why does the yellow‐eyed ensatina have yellow eyes? Batesian mimicry of Pacific newts (genus *Taricha*) by the salamander *Ensatina eschscholtzii xanthoptica* . Evolution, 62, 984–990. 18248632 10.1111/j.1558-5646.2008.00338.x

[ece311438-bib-0046] Lofts, B. (1964). Seasonal changes in the functional activity of the interstitial and spermatogenetic tissues of the green frog, *Rana esculenta* . General and Comparative Endocrinology, 4, 550–562. 14217824 10.1016/0016-6480(64)90064-4

[ece311438-bib-0047] Lunghi, E. , Monti, A. , Binda, A. , Piazzi, I. , Salvadori, M. , Cogoni, R. , Riefolo, L. A. , Biancardi, C. , Mezzadri, S. , & Avitabile, D. (2017). Cases of albinism and leucism in amphibians in Italy: New reports. Natural History Sciences, 4, 73–80.

[ece311438-bib-0048] Lynch, L. C. , & Blackburn, D. G. (1995). Effects of testosterone administration and gonadectomy on nuptial pad morphology in overwintering male leopard frogs, *Rana pipiens* . Amphibia‐Reptilia, 16, 113–121.

[ece311438-bib-0049] Miller, B. T. , Hall, E. M. , & Rollins‐Smith, L. A. (2018). Axanthism in the southern leopard frog, *Lithobates sphenocephalus* (cope, 1886), (Anura: Ranidae) from the state of Tennessee, USA. Herpetology Notes, 11, 601–602.

[ece311438-bib-0050] Mitchell, J. C. , & Church, D. R. (2002). Leucistic marbled salamanders (*Ambystoma opacum*) in Virginia. Herpetological Review, 29, 229–230.

[ece311438-bib-0051] Nielsen, H. I. (1978). The effect of stress and adrenaline on the color of *Hyla cinerea* and *Hyla arborea* . General and Comparative Endocrinology, 36, 543–552. 571381 10.1016/0016-6480(78)90094-1

[ece311438-bib-0052] Nilsson, S. H. , Aspengren, S. , & Wallin, M. (2013). Rapid color change in fish and amphibians–function, regulation, and emerging applications. Pigment Cell and Melanoma Research, 26, 29–38. 23082932 10.1111/pcmr.12040

[ece311438-bib-0053] Noonan, B. P. , & Comeault, A. A. (2009). The role of predator selection on polymorphic aposematic poison frogs. Biology Letters, 5, 51–54. 19019778 10.1098/rsbl.2008.0586 PMC2657764

[ece311438-bib-0054] Poulton, E. B. (1890). The colours of animals: Their meaning and use. Kegan Paul, Trench, Trubner.

[ece311438-bib-0055] Richards, C. M. (1982). The alteration of chromatophore expression by sex hormones in the Kenyan reed frog, *Hyperolius viridiflavus* . General and Comparative Endocrinology, 46, 59–67. 7060936 10.1016/0016-6480(82)90163-0

[ece311438-bib-0056] Robinson, M. (2022). “I've seen only one in my life”: The blue tree frog mystery. Australian Geographic.

[ece311438-bib-0057] Rodríguez, A. , Mundy, N. I. , Ibáñez, R. , & Pröhl, H. (2020). Being red, blue and green: The genetic basis of coloration differences in the strawberry poison frog (*Oophaga pumilio*). BMC Genomics, 21, 301. 32293261 10.1186/s12864-020-6719-5 PMC7158012

[ece311438-bib-0058] Rojas, B. (2017). Behavioural, ecological, and evolutionary aspects of diversity in frog colour patterns. Biological Reviews, 92, 1059–1080. 27020467 10.1111/brv.12269

[ece311438-bib-0059] Rojas, B. , & Endler, J. A. (2013). Sexual dimorphism and intra‐populational colour pattern variation in the aposematic frog *Dendrobates tinctorius* . Evolutionary Ecology, 27, 739–753.

[ece311438-bib-0060] Rowland, H. M. , Cuthill, I. C. , Harvey, I. F. , Speed, M. P. , & Ruxton, G. D. (2008). Can't tell the caterpillars from the trees: Countershading enhances survival in a woodland. Proceedings of the Royal Society B: Biological Sciences, 275, 2539–2545. 10.1098/rspb.2008.0812 PMC2605806 18700207

[ece311438-bib-0061] Rudh, A. , & Qvarnström, A. (2013). Adaptive colouration in amphibians. Seminars in Cell and Developmental Biology, 24, 553–561. 23664831 10.1016/j.semcdb.2013.05.004

[ece311438-bib-0062] Sanabria, E. A. , Quiroga, L. B. , & Laspiur, A. (2010). First record of partial albinism and scoliosis in *Odontophrynus occidentalis* tadpoles (Anura: Cycloramphidae). Brazilian Archives of Biology and Technology, 53, 641–642.

[ece311438-bib-0063] Sazima, I. (1974). An albino hylid frog, *Phrynohyas mesophaea* (Hensel). Journal of Herpetology, 8, 264–265.

[ece311438-bib-0065] Sillman, A. , Govardovskii, V. , Röhlich, P. , Southard, J. , & Loew, E. (1997). The photoreceptors and visual pigments of the garter snake (*Thamnophis sirtalis*): A microspectrophotometric, scanning electron microscopic and immunocytochemical study. Journal of Comparative Physiology A, 181, 89–101. 10.1007/s003590050096 9251253

[ece311438-bib-0066] Smith‐Gill, S. J. , Richards, C. M. , & Nace, G. W. (1972). Genetic and metabolic bases of two “albino” phenotypes in the leopard frog, *Rana pipiens* . Journal of Experimental Zoology, 180, 157–167. 4623610 10.1002/jez.1401800203

[ece311438-bib-0067] Sumida, M. , Islam, M. M. , Igawa, T. , Kurabayashi, A. , Furukawa, Y. , Sano, N. , Fujii, T. , & Yoshizaki, N. (2016). The first see‐through frog created by breeding: Description, inheritance patterns and dermal chromatophore structure. Scientific Reports, 6, 24431. 27080918 10.1038/srep24431 PMC4832234

[ece311438-bib-0068] Sztatecsny, M. , Preininger, D. , Freudmann, A. , Loretto, M.‐C. , Maier, F. , & Hödl, W. (2012). Don't get the blues: Conspicuous nuptial colouration of male moor frogs (*Rana arvalis*) supports visual mate recognition during scramble competition in large breeding aggregations. Behavioral Ecology and Sociobiology, 66, 1587–1593. 23162205 10.1007/s00265-012-1412-6 PMC3496481

[ece311438-bib-0069] Taboada, C. , Brunetti, A. E. , Lyra, M. L. , Fitak, R. R. , Faigón, S. A. , Ron, S. R. , Lagorio, M. G. , Haddad, C. F. , Lopes, N. P. , & Johnsen, S. (2020). Multiple origins of green coloration in frogs mediated by a novel biliverdin‐binding serpin. Proceedings of the National Academy of Sciences, 117(18), 574–581. 10.1073/pnas.2006771117 PMC7414155 32661155

[ece311438-bib-0070] Tang, Z. J. , Lue, S. I. , Tsai, M. J. , Yu, T. L. , Thiyagarajan, V. , Lee, C. H. , Huang, W.‐T. , Weng, C.‐F. , & Weng, C. F. (2014). The hormonal regulation of color changes in the sexually dichromatic frog *Buergeria robusta* . Physiological and Biochemical Zoology, 87, 397–410. 24769704 10.1086/675678

[ece311438-bib-0071] Taylor, J. D. , & Bagnara, J. T. (1972). Dermal chromatophores. American Zoologist, 12, 43–62.

[ece311438-bib-0072] Thongproh, P. , Chunskul, J. , Sringurngam, Y. , Waiprom, L. , Makchai, S. , Cota, M. , Duengkae, P. , Duangjai, S. , Hasan, M. , Chuaynkern, C. , & Chuaynkern, Y. (2022). A new species of the genus *Hoplobatrachus* Peters, 1863 (Anura, Dicroglossidae) from northwestern Thailand. Agriculture and Natural Resources, 56, 1135–1152.

[ece311438-bib-0073] Trullas, S. C. , van Wyk, J. H. , & Spotila, J. R. (2007). Thermal melanism in ectotherms. Journal of Thermal Biology, 32, 235–245.

[ece311438-bib-0074] Turner, G. S. (2017). A hypomelanistic Roth's tree frog *Litoria Rothii* from North Queensland. Queensland Naturalist, 55, 22–28.

[ece311438-bib-0075] Vershinin, V. (2004). Frequency of iris depigmentation in urban populations of *Rana arvalis* frogs. Russian Journal of Ecology, 35, 58–62.

[ece311438-bib-0076] Vitt, L. J. , & Caldwell, J. P. (2013). Herpetology: An introductory biology of amphibians and reptiles. Academic press.

[ece311438-bib-0077] Wilkens, H. (1988). Evolution and genetics of epigean and cave *Astyanax fasciatus* (Characidae, Pisces) support for the neutral mutation theory. In Evolutionary biology (Vol. 23, pp. 271–367). Springer.

